# Application of bundle management strategies in reducing local sterile abscesses following leuprolide acetate microsphere injections

**DOI:** 10.3389/fped.2026.1752815

**Published:** 2026-03-18

**Authors:** Jinfang Yuan, Linghui Li, Ying Zhao, Tongyan Han, Xinli Wang, Jiaqi Wang

**Affiliations:** Department of Pediatric, Peking University Third Hospital, Beijing, China

**Keywords:** bundle management strategies, central precocious puberty, injection-site adverse reactions, leuprolide acetate microspheres, local sterile abscess

## Abstract

**Objective:**

To investigate the effect of bundle management strategies on reducing the incidence of local sterile abscesses following leuprolide acetate microsphere injections.

**Methods:**

A single-center, intervention evaluation with a historical control study was conducted. Female children treated with leuprolide acetate microspheres for central precocious puberty (CPP) at the Pediatric Outpatient Department of Peking University Third Hospital from January 2016 to December 2024 were included as study participants. Patients from January 2016 to December 2018 served as the control group (277 individuals, 5,023 injections), during which high-risk factors for sterile abscesses were identified and bundle improvement strategies were formulated for continuous quality improvement. Patients from January 2019 to December 2024 were assigned to the intervention group (994 individuals, 13,498 injections). The control group received conventional management protocols, while the intervention group implemented a bundle management scheme encompassing medical and nursing staff training, standardized procedures, use of standardized tools, health education, and follow-up management. The incidence of local sterile abscesses was compared between the two groups.

**Results:**

After implementation of the bundle management strategies, the incidence of sterile abscesses in the control group was 5.1% (14/277) by patient count and 3.2‰ (16/5,023) by injection count, which decreased to 0.6% (6/994) and 0.4‰ (6/13,498) in the intervention group, respectively, with statistically significant intergroup differences (*P* < 0.001 for both).

**Conclusion:**

Implementation of bundle management strategies significantly reduces the incidence of local sterile abscesses after leuprolide acetate microsphere injections, enhances nurses' standardized operational proficiency and professional competence, alleviates patient suffering, improves treatment adherence, and warrants clinical adoption.

## 1 Introduction

Leuprolide acetate microspheres for injection are a commonly used gonadotropin-releasing hormone agonist (GnRHa) medication for treating idiopathic central precocious puberty (CPP) in children (1–4); however, local adverse reactions following the injection of this medication remain a concern that cannot be overlooked. Literature reports indicate that during treatment with leuprolide acetate microspheres, the incidence of local adverse reactions at the injection site reaches 34%, with sterile abscesses accounting for 8% (5), which not only increases patient discomfort but may also lead to treatment interruption. Although some studies report the incidence of injection-site adverse reactions and management measures for sterile abscesses (6), there is limited reporting on strategies to reduce local sterile abscesses of the skin.

The occurrence of local sterile abscesses following leuprolide acetate microsphere injections results from multiple factors, and conventional interventions are insufficient to provide comprehensive coverage. Bundle management strategies represent a comprehensive management approach that employs multiple evidence-based practices to enhance the quality of healthcare delivery. Widely utilized in healthcare settings, these strategies aim to prevent and manage various health conditions and, compared to routine care, can reduce the risk of adverse outcomes (7, 8). This study retrospectively analyzes the application of bundle management strategies in managing local sterile abscesses induced by leuprolide acetate microsphere injections and evaluates the effectiveness of this approach in clinical practice.

## 2 Materials and methods

### 2.1 Study participants

This study was a single-center, retrospective case-control study. Inclusion criteria were: (1) Attendance at the Pediatric Outpatient Department of our hospital from January 2016 to December 2024; (2) Meeting the diagnostic criteria for CPP as outlined in the Expert Consensus on the Diagnosis and Treatment of Central Precocious Puberty (9); (3) Female sex; (4) Receipt of regular treatment with the leuprorelin acetate (Enantone, Livzon Pharmaceutical Group Inc, Zhuhai, China) and administered subcutaneously once monthly; and (5) Receipt of regular injections at our hospital outpatient department. Exclusion criteria were: (1) Comorbid immunodeficiency disorders (e.g., primary immunodeficiency, long-term use of immunosuppressants); (2) Preexisting skin conditions at the injection site (e.g., eczema, dermatitis); (3) Concurrent infectious diseases during treatment (e.g., pneumonia, sepsis); (4) Other drug injections within 24 h before or after treatment; and (5) Missing clinical records. Patients were assigned to control or intervention groups based on visit dates, according to the inclusion and exclusion criteria. Children who received leuprolide acetate microsphere injections from January 2016 to December 2018 comprised the control group, and those who received injections from January 2019 to December 2024 constituted the intervention group. The study was approved by our hospital Ethics Committee (approval number: 20250529-03014-0019) and was conducted in strict accordance with relevant guidelines and regulations; all procedures adhered to the ethical principles of the Declaration of Helsinki.

### 2.2 Study methods

#### 2.2.1 Intervention methods for the control group

Conventional intervention measures were employed, including: (1) adherence to hand hygiene; (2) use of a dedicated suspending agent for reconstitution, injected along the vial wall, followed by horizontal rolling of the vial in the palm for 30 s and slow vertical inversion 5–6 times, to avoid vigorous shaking and foam formation; (3) strict adherence to aseptic technique and standardized disinfection of the injection site; (4) administration using a No. 7 subcutaneous needle (0.7 mm × 25 mm) at the lower border of the deltoid muscle; (5) rotation of injection sites at each administration to avoid repeated injections at the same location; and (6) no massaging of the injection site after administration.

#### 2.2.2 Intervention methods for the intervention group

Bundle management strategies were implemented in addition to conventional measures. The interventions, in addition to conventional measures, included: (1) establishment of a quality-improvement team in November 2018 comprising a chief physician specializing in endocrinology, an attending physician in endocrinology, a head nurse, and two supervising nurses. The head nurse served as team leader, responsible for coordinating and supervising the implementation of bundle improvement measures. Team members' responsibilities were clearly defined and included real-time problem analysis, discussion, and continuous improvement. (2) Literature review and retrospective analysis: Team members systematically reviewed relevant literature (5, 10, 11) and retrospectively collected and analyzed risk factors for sterile abscesses in the control group using medical record audits and telephone follow-up. (3) Analysis and determination of bundle management strategies: Through brainstorming, team members integrated literature findings with departmental realities, comprehensively discussing and analyzing risk factors for local sterile abscesses after leuprolide acetate microsphere injection from five perspectives—personnel, equipment, materials, methods, and environment—and constructed a bundle management strategy encompassing seven core components (Table 1). (4) Comprehensive training: All medical and nursing staff involved in diagnosis, administration, and follow-up underwent training on the bundle management strategy and achieved proficiency. Post-training evaluation included a theoretical examination (maximum score 100, passing score ≥80) and an operational assessment (reconstitution and injection procedure; passing score ≥85). Only participants who passed both evaluations were permitted to participate in the implementation to ensure standardized execution. (5) Quality control: The head nurse inspected nurses' adherence to standardized injection practices, the use of standardized tools, and the delivery of health education. The professional team leader supervised attending physicians' management of critical patient issues, serving as the first line of defense. Team members periodically reviewed the implementation of bundle improvement measures, analyzed and discussed problems, and promptly corrected them to promote correct execution and ensure standardization.

**Table 1:** Bundle management strategies for reducing local sterile abscesses following leuprolide acetate microsphere injections.

Category	Specific Measures
Medical and Nursing Staff Training	(1) Standardization of injection techniques (including key steps such as reconstitution, puncture, and needle withdrawal); (2) Patient health education content (adverse reaction identification and key home care points).
Physician Key Focus Points	(1) Assessment of the patient's history of local skin reactions post-injection; (2) Inspection of skin condition at the previous injection site, noting any inflammation or allergy; change formulation if present; (3) Emphasis t initial treatment on potential post-injection skin adverse reaction manifestations and precautions.
Standardized Procedures	(1) Pre-procedure patient assessment: Inquire about and examine skin condition at the injection site; review prior post-injection skin status; (2) Gently tap the vial before reconstitution to consolidate the drug powder at the vial base; (3) Aspiration of solvent: Use a 5 mL syringe with a No. 7 needle to aspirate solvent and inject along the vial wall; (4) Reconstitution: Place the vial flat in the palm, roll horizontally, then switch to vertical orientation and slowly invert up and down, avoiding vigorous shaking to achieve full suspension without foam formation; (5) Aspiration of drug suspension: Position the No. 7 needle tip at the vial base with the bevel fully apposed to the vial, aspirate the required dose to minimize air bubbles upon vial inversion; (6) Prohibit drug suspension standing post-reconstitution: Immediately replace with a No. 6 needle for subcutaneous injection in the child after aspiration; (7) Retain needle in place for 10 s post-injection; (8) Apply gentle pressure to the puncture site for 5–10 s after needle withdrawal, avoiding forceful compression.
Application of Standardized Tools	(1) Design and utilize standardized tools: Injection reminder card (see Table 5), including name, injection date, site, safety reminders, physician name, and triage desk telephone; (2) Card carried by caregiver; nurse verifies upon next injection; (3) Nurse cross-checks the reminder card with the patient information pre-injection and documents the injection site and signs post-injection.
Post-Injection Precautions Emphasis	(1) Prohibit water contact at the puncture site within 24 h (child prohibited from bathing or swimming within 24 h); (2) Prohibit scratching at the puncture site within 24 h; (3) Avoid vigorous activity of the limb at the puncture site within 24 h.
Health Education	(1) Use accessible language for drug-related knowledge and injection site selection; (2) Potential local adverse reaction manifestations post-injection, observation, and care; (3) Preventive measures for local adverse reactions post-injection, with emphasis on key home care points (e.g., initial management of redness and swelling); (4) Distribute illustrated health education manuals demonstrating adverse reaction observation methods.
Follow-Up	(1) Passive (patient contacts physician): Receive consultations via telephone, noted on a reminder card with response within 24 h; (2) Active follow-up (medical staff contacts patient): For children with initial reactions, telephone follow-up at 3 and 7 days post-injection; for those without reactions, follow-up once per monthly injection.
Management of Post-Local Sterile Abscess Occurrence	(1) Conservative treatment: Prioritize conservative observation upon sterile abscess occurrence; (2) Surgical intervention: Coordinate with relevant hospital departments for expedited (green channel) consultation.
Investigation and Analysis of Post-Local Sterile Abscess Occurrence	(1) Establish a quality improvement team (medical, nursing, pharmacy, and administrative management) to investigate and analyze sterile abscess cases; (2) Implement standardized forms to survey potential risk factors contributing to sterile abscess development.
Strategy Optimization	(1) Professional team leader and head nurse supervise and evaluate bundle improvement measures, correcting issues identified in practice; (2) Conduct regular group discussions to summarize and analyze bundle improvement measures, progressively revising the strategy to reduce sterile abscess incidence.

#### 2.2.3 Observational index

The study evaluated the effectiveness of bundle management strategies by analyzing the occurrence of sterile abscesses following leuprolide acetate microsphere injections in both groups, including incidence rates, associated risk factors, and severity grading.

Sterile abscesses were graded into three severity levels: (1) Mild: Localized induration or slight swelling only, range ≤5 cm, without pain or redness, no impact on activity, and no systemic symptoms such as fever; (2) Moderate: Prominent local swelling, range 5–10 cm, with mild pain or tenderness, possible pale red skin manifestation, no systemic symptoms, and no significant impact on activity; (3) Severe: Severe local swelling, range >10 cm, intense pain exacerbated by pressure, marked skin redness, possible fluctuation (indicating fluid accumulation), or accompanied by systemic symptoms such as fever or fatigue, impairing limb movement.

### 2.3 Statistical analysis

IBM Statistical Package for Social Sciences software version 25 was used for statistical analysis. For continuous variables, normality testing was conducted; those conforming to normal distribution were expressed as mean ± standard deviation (x¯ ± s), and differences between the case and control groups were compared using the t-test. Count data were expressed as “number of cases (rate)” and analyzed using contingency table tests, with *P* < 0.05 considered statistically significant.

## 3 Results

### 3.1 General data

This study retrieved data from the outpatient medical record system. From January 2016 to December 2024, 436,830 patients attended the Pediatric Outpatient Department of our hospital, of whom 5,800 were diagnosed with CPP, including 4,790 female children. Among them, 1,271 patients received regular leuprolide acetate microsphere treatment and injections, totaling 18,521 injections. The control group (January 2016 to December 2018) included 277 patients with 5,023 injections; the intervention group (January 2019 to December 2024) included 994 patients with 13,498 injections.

### 3.2 Incidence of sterile abscesses

**Figure 1:**
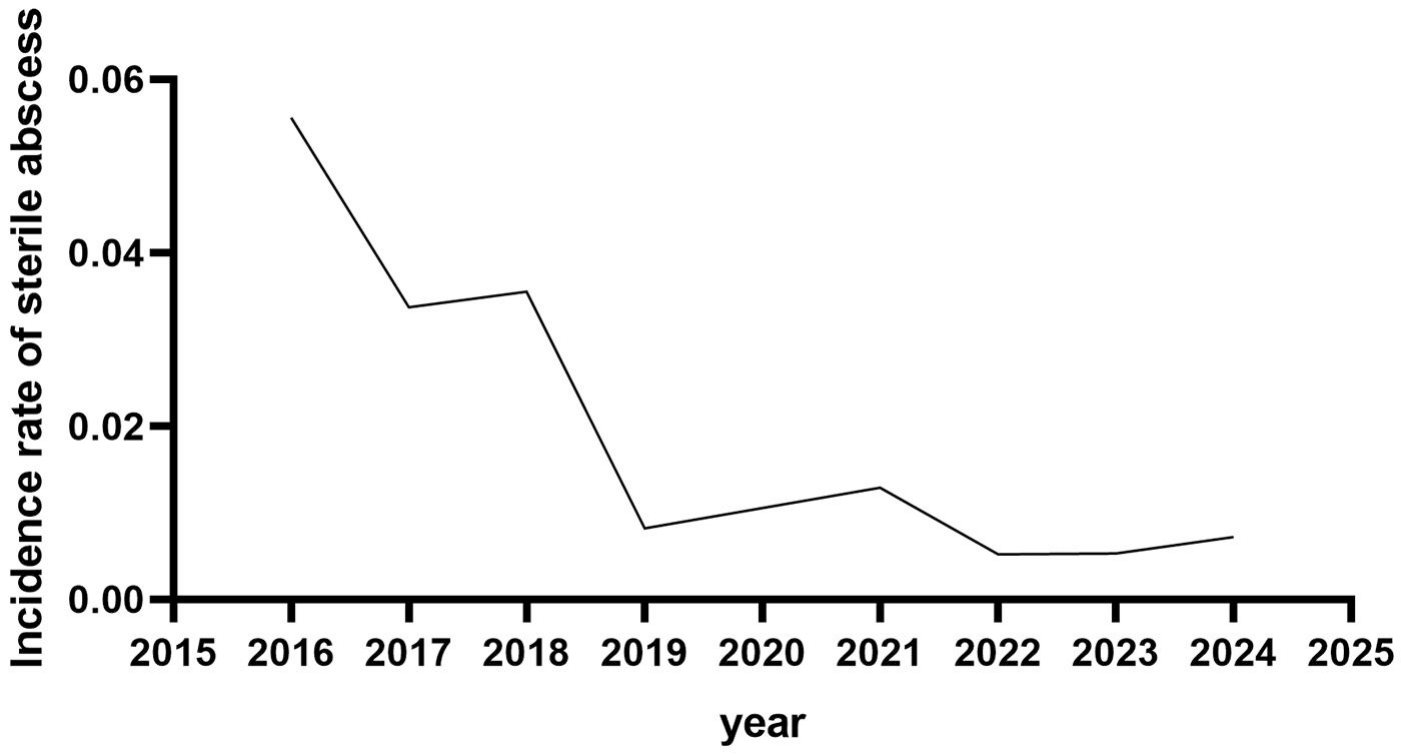
Annual incidence of sterile abscess.

A detailed review of medical records identified 20 cases of injection-site sterile abscesses. All children had previously been healthy, with no history of allergies or relevant family history; the annual incidence is shown in Figure 1. The control group had 14 cases with 16 sterile abscess events, mean age 10.1 ± 1.4 years, occurring at the 10.4 ± 4.9th injection, with incidence rates of 5.1% (14/277) by patient count and 3.2‰ (16/5,023) by injection count. The intervention group had 6 cases with six sterile abscess events, mean age 10.2 ± 2.0 years, occurring at the 9.0 ± 5.7th injection, with incidence rates of 0.6% (6/994) and 0.4‰ (6/13,498), respectively; intergroup differences were statistically significant (Table 2).

**Table 2:** Characteristics of children with aseptic abscesses.

Variables	Control Group (*n* = 14)	Intervention Group (*n* = 6)	F/*χ*^2^ Value	*P* Value
Age (years)	10.1 ± 1.4	10.2 ± 2.0	1.004	0.330
Number of Injections at Onset	10.4 ± 4.9	9.0 ± 5.7	0.000	0.996
Incidence (per person)	5.1% (14/277)	0.6% (6/994)	27.704	0.000
Incidence (per injection)	3.2‰ (16/5023)	0.4‰ (6/13498)	23.179	0.000

### 3.3 Risk factors associated with sterile abscess occurrence

Retrospective analysis of children with sterile abscesses in the control group identified potential causes: vigorous exercise within 24 h post-injection (basketball/running/soccer), local scratching, bathing within 24 h, and consecutive injections at the same site. Risk factors for sterile abscess occurrence were statistically compared between the control and intervention groups; details are presented in Table 3.

**Table 3:** Statistical analysis of risk factors associated with aseptic abscesses in children.

Risk Factors	Control Group (*n* = 16)	Intervention Group (*n* = 6)
Vigorous exercise within 24 h post-injection (basketball/running/soccer)	3	0
Local scratching	2	0
Bathing within 24 h (after injection)	2	0
Continuous injection at the same site	2	0
Unknown causes	7	6

### 3.4 Severity grading of sterile abscesses

Analysis of sterile abscess cases revealed 22 total events; the severity-grade distribution is shown in Table 4. Patients with mild sterile abscesses recovered within 2 days, moderate cases within 5–8 days, and severe cases (all presenting local pus discharge without systemic discomfort) recovered within 2 weeks, with no residual local scarring.

**Table 4:** Statistical analysis of severity grading of aseptic abscesses.

Severity Grade	Control Group (*n* = 16)	Intervention Group (*n* = 6)
Mild	6	3
Moderate	8	2
Severe	2	1

## 4 Discussion

Analyses from the FAERS database indicate that sterile abscesses represent one of the severe injection-site adverse events associated with GnRHa in the treatment of children with CPP (12). A limited number of large-sample studies exist regarding this issue. To our knowledge, this study provides one of the largest datasets on the incidence of aseptic abscesses in children with CPP to date. Research by Johnson SR and colleagues suggests that the mechanism of sterile abscess development in children with CPP treated with GnRHa is complex, potentially involving adverse reactions to biodegradable inert polymers, *in vivo* anti-GnRHa protein antibody production, and factors such as varying injection techniques and site selection (13–15). However, reports on reducing local sterile abscesses remain scarce. Bundle management strategies were first proposed by the U.S. Institute for Healthcare Improvement and involve integrating and implementing a series of evidence-based measures to address specific clinical conditions (16); the concurrent application of these measures yields greater efficacy than individual implementation (7, 17, 18). Under bundle management strategies, multiple measures are consolidated to maximize effect, with ongoing evaluation and continuous refinement.

Grounded in evidence-based practice and integrating the mechanisms of sterile abscess formation with risk factors identified in the control group of this study, a comprehensive bundle management strategy was developed spanning the pre-, intra-, and post-medication phases for pediatric patients. All injections were given via the subcutaneous route in our center. The incidence of aseptic abscess was not influenced by different injection methods. Pre-medication components included specialized training and assessment for medical and nursing staff, joint pre-administration evaluation, and health education; intra-medication measures focused on standardized injection techniques and the application of standardized reminder cards; post-medication encompassed post-injection precaution communication, combined active and passive follow-up, quality feedback, and dynamic strategy optimization. Results demonstrated a 5.1% sterile abscess incidence in the control group, which decreased to 0.6% following implementation of the bundle management strategy, conclusively showing that the full-process approach significantly mitigates the risk of local sterile abscesses after GnRHa injection.

### 4.1 Dual-layer defense via pre-administration medical-nursing evaluation in bundle management strategies

Deficient knowledge among parents and caregivers is one of the most commonly cited causes of medication errors by pediatricians and parents alike (19). In this study, joint medical-nursing pre-administration evaluation of pediatric patients under the full-process bundle management strategy in the intervention group constituted the first line of defense against sterile abscesses. Physicians conducted precise assessments tailored to the medication phase: for initial treatment, the focus was on injection-site skin condition, allergy history, and immune response, with communication of potential adverse reactions and post-injection precautions; for subsequent courses, prior injection-site local skin status was reviewed for pain, induration, or redness, with cause analysis and formulation adjustment if no clear trigger was identified to prevent recurrence. Nurses re-evaluated and reinforced these points before injection, establishing a second line of defense through duplicate assessment and communication to mitigate oversights from single-role evaluation and reduce risks due to inadequate assessment. The bundle management strategy thus establishes a dual-layer safety defense against the occurrence of sterile abscess through collaborative medical-nursing pre-administration evaluation.

### 4.2 Multidimensional reinforced education in bundle management strategies to heighten caregiver awareness

Studies indicate that while caregivers possess some capacity to manage local adverse reactions following leuprolide acetate microsphere injections in children with CPP, their relevant knowledge remains inadequate (10). Retrospective review of sterile abscess cases in the control group revealed risk factors including local scratching, vigorous activity, bathing, and consecutive same-site injections; notably, one child experienced three sterile abscesses, with two instances directly linked to bathing and vigorous exercise within 24 h after injection upon history tracing. After an abscess occurred, we adjusted the treatment. After approximately 8–10 intervals, some parents requested to resume the previous therapeutic regimen. In our clinical practice, most patients did not experience recurrent abscesses after reuse of the drug. This case is relatively unusual and fortunately reactions were relieved without any particular treatment. Accordingly, beyond distributing illustrated educational manuals and science-popularization videos, the bundle management strategy incorporated reinforced health education at multiple points—patient visits, physician prescribing, and pre- and post-nursing administration—with repeated emphasis on precautions to embed core post-injection guidelines into caregivers' and patients' behavioral memory for instinctive compliance. Practice confirms that this full-process, multidimensional, reinforced education effectively enhanced caregivers' knowledge mastery and prioritization during pediatric medication, resulting in a significant decline in the intervention group's sterile abscess incidence attributable to previously identified risk factors.

### 4.3 Standardized tools in bundle management strategies effectively mitigate repeated same-site injection risk

Medication administration in children with CPP is a protracted process requiring pediatric self-management, wherein medication management is pivotal to slowing chronic disease progression, reducing complications, lowering disability rates, enhancing quality of life, and decreasing healthcare costs (20). Risk factor analysis at our center indicates that administering consecutive injections at the same site increases the risk of sterile abscess development in children receiving GnRHa treatment for CPP. Given the prolonged interval between doses (every 1–3 months), some caregivers or children readily forget prior injection sites, increasing the likelihood of repeated same-site administration and thus elevating the risk of sterile abscess. In the control group of this study, two sterile abscess cases were directly attributable to parental forgetfulness of previous sites. A pediatric self-management mechanism was established within the intervention group's bundle management strategy to prevent adjacent injections at the same site, thereby alleviating local immune responses and reducing the incidence of adverse reactions through the design and application of a standardized tool—the injection reminder card (Table 5). Nurses completed the card after each injection, documenting the date, site, and operator, and affixed it to the prescription, enabling caregivers and medical staff to review prior injection details before the next administration while confirming the current date and site, thereby reducing the risk of repeated same-site injection.

**Table 5:** Injection reminder card.

Injection Number	Injection Date	Injection Site	Administering Nurse
01			
02			
03			
Please note the following: 1. Rotate injection sites, avoiding the same site as the previous injection whenever possible; 2. Avoid bathing, hot compresses, or vigorous exercise within 24 h post-injection; keep the injection site dry; 3. Do not rub, press, pat, or scratch the injection site; 4. Avoid vaccinations on the same day; 5. Avoid tight clothing and change undergarments frequently; 6. In case of cold, fever, or other discomfort, seek prompt medical attention and ensure full recovery before the next injection; 7. For concurrent immune system abnormalities such as rash, erythema, eczema, allergic dermatitis, pollen allergy, or lupus erythematosus, consult a specialist before deciding on injection.			

Consultation Telephone: 82264203.

### 4.4 Follow-up system in bundle management strategies minimizes the impact of adverse reactions

A retrospective clinical study by Zhang Tian et al. reported a sterile abscess incidence of 1.23% (4/326), with three cases requiring surgical incision and drainage (21); further reports note that multiple-site sterile abscesses during GnRHa treatment in children with CPP can cause severe scarring (13), underscoring that improper management of such local adverse reactions may impose additional health burdens on pediatric patients. In the intervention group of this study, passive and active follow-up channels enabled medical staff to promptly ascertain local adverse reaction occurrence at injection sites, accurately evaluate resolution or progression, and guide caregivers on appropriate responses, subsequent measures, or the need for in-person visits. Dedicated follow-up personnel recorded details comprehensively for reporting, with team discussion and cause analysis informing continuous strategy refinement as needed. This combined active-passive follow-up intervention effectively confined the impact of adverse reactions to the minimum possible range. All sterile abscess cases in both the control and intervention groups exhibited spontaneous resolution of local redness and swelling after pus discharge, with abscess rupture healing without surgical intervention or residual scarring. However, sterile abscess incidence declined markedly in the intervention group. Collectively, constructing a robust follow-up system with dedicated personnel for timely monitoring, guidance, and intervention of pediatric injection-site local adverse reactions during GnRHa therapy constitutes an effective and essential measure to mitigate adverse impacts and ensure treatment safety, offering greater clinical value than reliance on spontaneous resolution.

This study has some limitations. Sterile abscess occurrence after GnRHa injection is influenced by multiple factors; both the control and intervention groups included cases without a clearly documented etiology, and prolonged intervals to telephone follow-up precluded avoidance of recall bias or refusal to provide information by children or families, potentially leading to inaccuracies in some medication records. Therefore, the bundle management strategy proposed herein requires ongoing clinical application and optimization to further reduce the incidence of such adverse reactions.

In summary, the practice of bundle management strategies in GnRHa treatment of CPP children significantly reduces local sterile abscess incidence, alleviates pediatric suffering, ensures medication safety while enhancing adherence, minimizes risks of medical disputes, demonstrates favorable clinical application, and merits widespread promotion.

## Data Availability

The raw data supporting the conclusions of this article will be made available by the authors, without undue reservation.
